# A characterization of dynamic resistance training‐induced skeletal muscle adaptations in hypertensive rats

**DOI:** 10.14814/phy2.70516

**Published:** 2025-10-02

**Authors:** Amanda Aparecida de Araujo, Danielle da Silva Dias, Agnelo Neves Alves, Nathalia Bernardes, Rui Curi, Nilsa Regina Damaceno‐Rodrigues, Elia Garcia Caldini, Raquel Agnelli Ferreira‐Mesquita, Maria Claudia Irigoyen, Kátia De Angelis

**Affiliations:** ^1^ Postgraduate Program in Rehabilitation Sciences Universidade Nove de Julho (UNINOVE) Sao Paulo Brazil; ^2^ Laboratory of Exercise Physiology Federal University of Sao Paulo (UNIFESP) Sao Paulo Brazil; ^3^ Laboratory of Cell Biology, Medical School University of Sao Paulo Sao Paulo Brazil; ^4^ Human Movement Laboratory Sao Judas Tadeu University (USJT) Sao Paulo Brazil; ^5^ Department of Physiology and Biophysics, Institute of Biomedical Sciences University of São Paulo Sao Paulo Brazil; ^6^ Heart Institute (InCor) Medical School, University of Sao Paulo Sao Paulo Brazil

**Keywords:** dynamic resistance training, hypertension, muscle fiber type, sex differences

## Abstract

Resistance training (RT) is commonly recommended to increase muscle strength and to complement aerobic training for managing hypertension. However, the effects of RT on muscle adaptations in hypertensive individuals are not well understood. This study evaluated RT‐induced muscular changes in spontaneously hypertensive rats (SHRs). Thirty‐two adult SHRs were divided into four groups: sedentary females (SF), trained females (TF), sedentary males (SM), and trained males (TM). The trained groups underwent moderate‐intensity dynamic RT 5 days per week for 8 weeks. Muscle assessments included measuring phosphofructokinase (PFK) activity, myosin heavy chain (MHC) expression, and fiber type. Blood pressure (BP) was measured directly. The results showed increased type IIB fibers in the trained females (TF) and more intermediate fibers in the trained males (TM). Trained rats displayed higher PFK activity and MHC IIB expression in the plantaris muscle. There were positive correlations between the maximal load test (MLT) and MHC IIB expression in all groups, MLT and intermediate fibers in males, and MLT and IIB fibers in females. No significant changes in BP were observed following the training period. These results suggest that RT promotes beneficial muscle adaptations in both sexes, with some sex‐specific differences in fiber composition. Although BP was unchanged, the findings support RT as a strategy to improve functional capacity in this population.

## 1 INTRODUCTION

Resistance training (RT) is typically recommended to enhance muscle strength and hypertrophy. In recent years, most international guidelines have also recommended RT as a complement to aerobic training to improve skeletal muscle strength, control body composition, and assist in the management of cardiovascular diseases such as hypertension (James et al., 2014; Mozaffarian et al., 2015). While the antihypertensive effects of aerobic training are well established, the role of RT in lowering blood pressure (BP) remains less clear, with inconsistent evidence regarding its efficacy. Nevertheless, RT has been increasingly incorporated into clinical guidelines because of its additional health benefits, including improved muscle function, metabolic control, and overall quality of life (MacDonald et al., 2016; Williams et al., 2007). This underscores the importance of better understanding its specific effects on BP and the underlying mechanisms involved, particularly in hypertensive individuals.

To contribute to this knowledge, our research group developed a standardized maximal load test (MLT) protocol for rats at increased cardiovascular risk. This approach allows for precise RT prescription in terms of load, ensures training effectiveness, and avoids the use of painful stimuli or food‐based motivation (Sanches et al., 2014). Furthermore, when applying this MLT‐based RT protocol at guideline‐recommended intensities (Pescatello et al., 2004), we demonstrated BP reduction and sympathetic tonus attenuation (Shimojo et al., 2015), alongside better oxidative stress control (da Palma et al., 2016) in hypertensive ovariectomized female rats.

Despite these promising findings, the effects of RT on BP in isolation—that is, without confounding comorbidities—remain underexplored, particularly when considering potential sex differences. Current hypertension management guidelines do not distinguish between sexes, although evidence suggests that women may be at greater cardiovascular risk from elevated BP than men. Gillis and Sullivan (2016) highlighted this disparity; yet most experimental models focus solely on males, potentially overlooking sex‐specific responses (Clayton & Collins, 2014; Gillis & Sullivan, 2016).

Interest in sex differences in cardiovascular health is growing, but relatively little is known about how these differences influence skeletal muscle, which may affect both disease progression and responses to nonpharmacological interventions (Haizlip et al., 2015). For instance, Lundsgaard and Kiens (2014) reported sex‐based differences in muscle fiber type distribution in the vastus lateralis. Experimental studies have also shown sex‐related variations in myosin heavy chain (MHC) expression in the soleus muscle (Haizlip et al., 2015) and in mitochondrial proteins following endurance training (Landen et al., 2023). In hypertensive male rats and humans, skeletal muscle exhibits a shift toward fatigue‐resistant fibers and decreased strength as hypertension progresses (Atrakchi et al., 1994; Ben Bachir‐Lamrini et al., 1990; Juhlin‐Dannfelt et al., 1979).

Given the ongoing debate about RT's impact on ambulatory BP and its growing clinical use for its broader functional benefits, further investigation is warranted. In particular, it is essential to clarify the muscular adaptations to RT across sexes in the context of hypertension. Therefore, the present study aimed to evaluate the muscular adaptations induced by moderate‐intensity dynamic RT in male and female spontaneously hypertensive rats (SHRs).

## 2 MATERIALS AND METHODS

### 2.1 Ethics statement

All surgical procedures and protocols were in accordance with the recommendations in the Guide for the Care and Use of Laboratory Animals of the National Institutes of Health (National Research Council (US) Committee for the Update of the Guide for the Care and Use of Laboratory Animals, 2011) and were approved by the Nove de Julho University Ethical Committee (protocol 0010/2015).

### 2.2 Animals and groups

Third‐two adult male and female SHR (16 males), 3 months old, were obtained from the Biotery of Nove de Julho University. The animals received freely available standard laboratory chow and water and were housed in a controlled room temperature (22°C) and kept under a controlled 12‐h light–dark cycle. Animals were clinically evaluated once a day, and body weight was measured weekly. Rats were assigned into four groups (*n* = 6–8 each): sedentary and trained female (SF and TF) and sedentary and trained male (SM and TM).

### 2.3 Evaluation of the estrous cycle

The characterization of each phase of the cycle was based on the proportion of three types of cells in the vaginal secretion: epithelial, corneified, and leukocytes (Marcondes et al., 2002). Vaginal secretion was collected with a plastic pipette containing 10 μL of saline solution introduced superficially into the vagina of the rat. This drop of saline solution was collected and placed on a glass slide for observation under an optical microscope (magnification of 400×). All evaluations were performed in the non‐ovulatory phases (diestrous and metaestrus) of the estrous cycle.

### 2.4 Maximal load test and resistance exercise training

The groups were adapted to a ladder adapted for rats, 104.4 cm high (three consecutive climbs), 51 vertical steps spaced 2.0 cm apart, and a rectangular base (21.3 × 40 × 3 cm) with 4 support feet. The steps had a 105° incline and a small rat cage at the top (31.7 × 19.3 × 21 cm) that was covered with a cloth to create a dark environment for the animal to rest between climbs.

A maximum load test was performed in the first week, in the 4th week, and at the end of the 8th week protocol. It consisted of an initial load of 75% of the body weight, which was attached to the base of the rat tail. The load was progressively incremented by 45% of the body weight in subsequent climbs, as previously described (Sanches et al., 2014).

**FIGURE 1:**
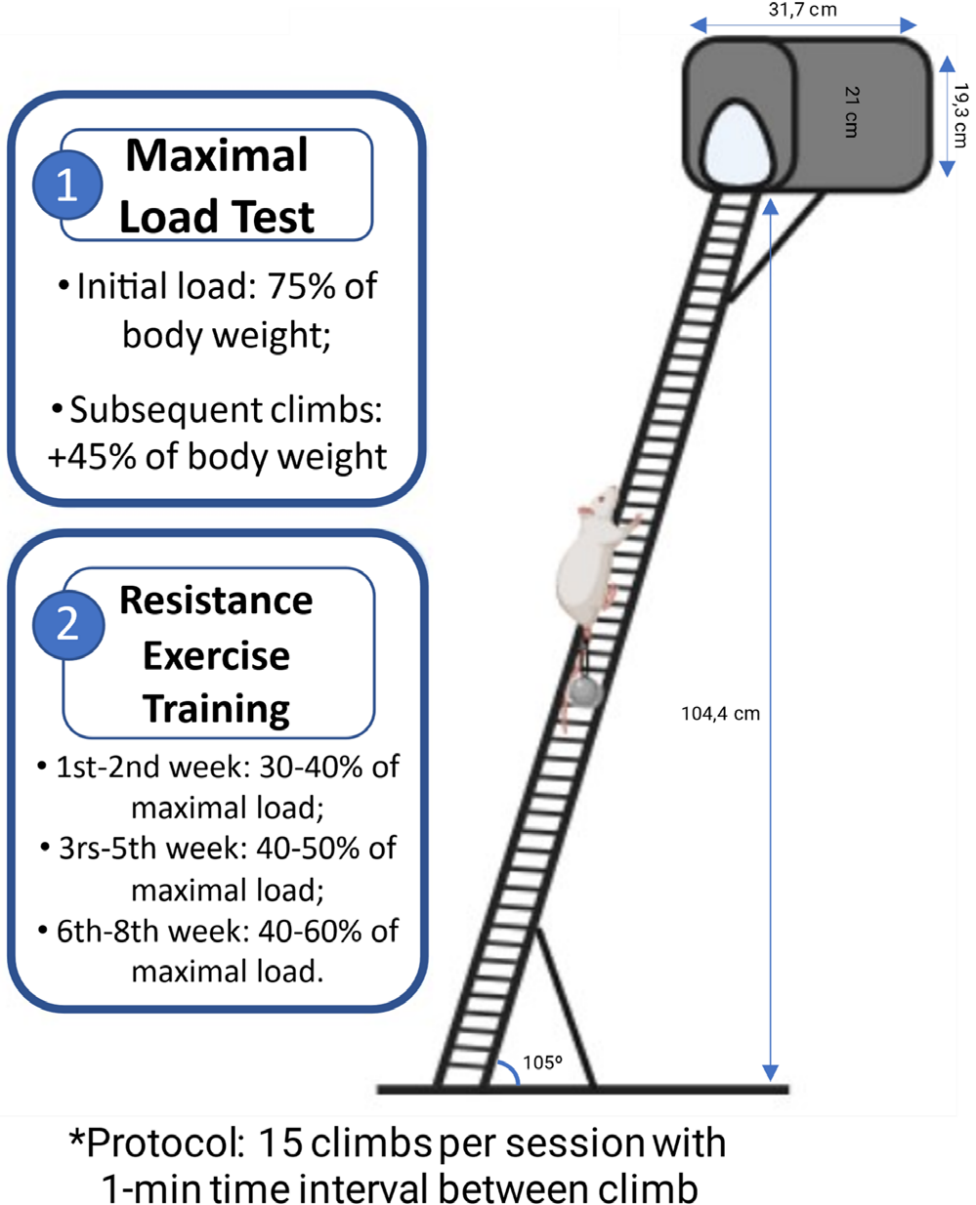
Illustration of the ladder adapted for rats used in this study. (1) Summary of the maximal load test; and (2) Summary of the dynamic resistance training.

The RT was then performed using the normalized value of the individual maximal load (load of the last complete climb/body weight) for each rat and was adjusted weekly according to the body weight of the animal. Resistance exercise was performed 5 days per week for 8 weeks at moderate intensity (1st–2nd week: 30%–40% of maximal load; 3rd–5th week: 40%–50% of maximal load; 6th–8th week: 40%–60% of maximal load). The rats performed 15 climbs per session with a 1‐min time interval between climbs as previously described (Figure 1) (Sanches et al., 2014).

### 2.5 Evaluation of the maximum oxygen consumption (VO_2_ max)

In order to perform the protocol of evaluation of maximum oxygen consumption, the animals were individually positioned in the metabolic box on the treadmill, where the rest values were initially collected. The observation time was approximately 30 min. After this time, the stress test was immediately initiated and consisted of subjecting the animal to running on the treadmill with an initial velocity of 0.3 km/h for 3 min, this load being increased by 0.3 km/h every 3 min until the animal to exhaustion. The maximum physical capacity assessment was performed using a respirometry open system. The metabolic determination of oxygen consumption was according to the method described by Brooks and White (1978). VO_2_ max was evaluated by means of a metabolic box connected to an oxygen sensor (O_2_ analyzer, AVS projects), which continuously analyzes samples of expired oxygen fractions (FeO_2_), as well as the values of environmental oxygen concentrations (FiO_2_). The VO_2_ values of each animal were calculated by the following mathematical formula: VO_2_ (mlO_2_. kg^−1^. min^−1^) = VE (FiO_2_−FeO_2_)/PC, where: VE: Suction pump flow rate (mL/min); FiO_2_: Inspired fraction of O_2_; FeO_2_: Expired fraction of O_2_; PC: Body weight of the animal (g). The VO_2_ reserve max. was obtained by the equation: VO_2_ reserve max = VO_2_ max−VO_2_ at rest.

### 2.6 Cardiovascular measurements

At the end of the RT protocol, rats were anesthetized with ketamine (90 mg/kg) and xylazine (12 mg/kg), and a polyethylene‐tipped Tygon cannula filled with heparinized saline was implanted into the carotid artery for direct measurements of AP. During experiments, rats received food and water ad libitum; they remained conscious in their cages and could move freely during the hemodynamic experiments. The arterial cannula was connected to a transducer (Blood Pressure XDCR, Kent© Scientific), and blood pressure signals were recorded for a 30‐min period using a microcomputer equipped with an analog‐to‐digital converter (WINDAQ, 2Kz, DATAQ Instruments). The recorded data were analyzed on a beat‐to‐beat basis to quantify changes in mean blood pressure (MBP).

### 2.7 Euthanasia

All animals were euthanized after hemodynamic measurements by decapitation, preceded by sedation with ketamine and xylazine. The soleus and plantaris muscles were carefully collected.

### 2.8 Measurement of citrate synthase (CS) in soleus muscles and phosphofructokinase (PFK) in plantaris muscle

To determine citrate synthase activity, we used an extraction buffer containing 0.5 mM Tris–HCl and 1 mM EDTA, pH 7.4, and an assay buffer containing 100 mMDTNB (0.2 mM), acetyl‐CoA (0.1 mM), and Triton X‐100 (0.1% v/v), pH 8.1. The reaction was initiated by the addition of 50 μL oxaloacetic acid (10 mM final concentration) and absorbance measurement at 412 nm (Alp et al., 1976).

PFK activity was determined using the method described by Opie and Newsholme (1967). This method evaluates the oxidation of reduced nicotinamide adenine dinucleotide phosphate. The extraction buffer consisted of Tris–HCl (50 mM), EDTA (1 mM), MgCl_2_ (5 mM), and β‐mercaptoethanol (5 mM) at pH 7.4. For determination of PFK activity, 50 μL of sample was added to 900 μL of assay buffer containing Tris–HCl (0,4 mM) and MgCl2 6H_2_O (5 mM) at pH 7.4. The mixture assay contained 1 mL of assay buffer, ATP (200 mM) (Sigma–Aldrich, St. Louis, MO, USA, Catalog number: A2383), NADH (13 mM) (ICN Biomedicals, Aurora, OH, USA), AMP (100 mM), αglycerol phosphate dehydrogenase (5 μg/mL), triose phosphate dehydrogenase (5 μg/mL), and aldolase (50 μg/mL). The reaction was initiated by the addition of 50 μL fructose 6‐phosphate (20 mM) (Sigma–Aldrich, St. Louis, MO, USA, Catalog number: 344342) and absorbance was determined by spectrophotometry at 340‐nm wavelength, at 25°C, for 10 min. The extraction medium for glucose‐6‐phosphate dehydrogenase (G6PDH) contained 50 mM Tris–HCl and 1 mM EDTA. The final pH value was 8.0. G6PDH was assayed by following the changes at 340 nm.

### 2.9 Real‐time quantitative polymerase chain reaction

Total RNA was isolated from the plantaris and soleus muscles using cold Trizol Reagent (Invitrogen, CA, USA, Catalog number: 15596026), following the manufacturer's instructions. RNA quantity and integrity were assessed in a NanoDrop 2000 spectrophotometer (Thermo Scientific, USA) and 1% agarose gel electrophoresis stained with ethidium bromide. cDNA synthesis was performed with 1 μg of total RNA and a High‐Capacity cDNA Reverse Transcription Kit (Applied Biosystems, USA, Catalog number: 4368814) using a Veriti® Thermal Cycler (Applied Biosystems, USA). All samples received DNase I to avoid DNA contamination. RT‐qPCR was performed using a 7500 Fast Real‐Time PCR System (Applied Biosystems, USA) and SYBR® Green PCR Master Mix (Applied Biosystems, USA, Catalog number: 4309155). The thermal cycling conditions were 50°C for 2 min, 95°C for 10 min, followed by 40 cycles at 95°C for 15 s and 60°C for 1 min. The primer oligonucleotides (forward and reverse primers) specific for MHC I and MHC IIb were used to perform this procedure. For normalization of the data, primers were used for GAPDH, and for the comparison between the data between the groups (control and treated), arbitrary units were calculated as follows (Mesquita‐Ferrari et al., 2015):

$$\text{Arbitrary unit}=2-\text{Delta}-\text{Delta}-\mathrm{Ct}\;\left(\text{DDCt}\right)$$

Being DDCt = DCT (Delta‐Ct) sample−DCt control.

### 2.10 Muscle fiber‐typing

The soleus and plantaris muscles were harvested and immediately frozen in isopentane and liquid nitrogen to correctly preserve the tissue, eliminating possible bias, and stored in −80 freezer. Frozen muscles (4–7 for each group) were cut into 10 μm cross‐sections through the proximal to distal region using a cryostat (Cryostat Microm HM505E, Microm International GmbH, Walldorf, Germany). Muscle sections were then incubated for myofibrillar ATPase activity after alkali (ATPase, pH 10.3) or acid preincubation (ATPase, pH 4.6) (Bacurau et al., 2009). The myosin ATPase reaction was used to identify muscle‐type fibers. Type I fibers reacted deeply after acid preincubation at pH 4.6 and lightly after alkali preincubation at pH 10.3. The inverse occurred with type II muscle fibers. Fiber typing was evaluated in whole muscles at 200 magnification and further analyzed on a digitizing unit connected to a computer (Image Pro‐plus; Media Cybernetic, Silver Spring, MD, USA). The total number of each fiber type was counted to calculate the numerical fiber‐type composition in soleus and plantaris. In plantaris, the fibers that could not be clearly identified as IIA, IIX, or IIB were classified as intermediate (they presented as a light stain at pH 10.3 and a small area). All analyses were conducted by a single observer (Amanda Aparecida de Araujo).

### 2.11 Statistical analysis

Data are expressed as means ± standard deviation. Data were tested to evaluate homogeneity. One‐way ANOVA or two‐way ANOVA was used as required, followed by the Student–Newman–Keuls test to compare groups. The significance level was established at *p* < 0.05. Pearson correlates with linear regression were also applied, considering a 95% confidence interval.

## 3 RESULTS

The female groups had lower body weights and less white adipose tissue than the male groups. All groups gained weight by the end of the protocol. TF had less white adipose tissue than SF (Table 1).

**TABLE 1:** Body weight at the beginning (Initial) and after 8 weeks (Final) of the protocol, white adipose tissue normalized by body weight, plantaris, and soleus muscle weight and mean blood pressure after 8 weeks of protocol.

	SM	TM	SF	TF	*p*
Body weight (g)					
Initial	241 ± 15	253 ± 8	162 ± 11	159 ± 9	<0.0001
Final	306 ± 18^‡^	309 ± 19^‡^	190 ± 16^‡^ ^,^ ^#^	186 ± 6^‡^ ^,^ *	
White adipose tissue (mg/g of body weight)	8.03 ± 0.92	6.37 ± 0.76^#^	5.09 ± 0.36^#^	2.87 ± 0.33* ^,^ ^†^	<0.0001
Plantaris weight (mg)	201 ± 38	263 ± 26^#^	152 ± 27	182 ± 24^†^	<0.0001
Soleus weight (mg)	112 ± 12	115 ± 8^#^	84 ± 11	82 ± 9^†^	<0.0001
Mean blood pressure (mmHg)	185 ± 21	186 ± 10	172 ± 15	173 ± 18	0.2752

Abbreviations: SF, sedentary female; SM, sedentary male; TF, trained female; TM, trained male.

^‡^ Versus initial in the same group.

^#^ *p* < 0.05 versus SM.

* *p* < 0.05 versus TM.

^†^ *p* < 0.05 versus SF.

**FIGURE 2:**
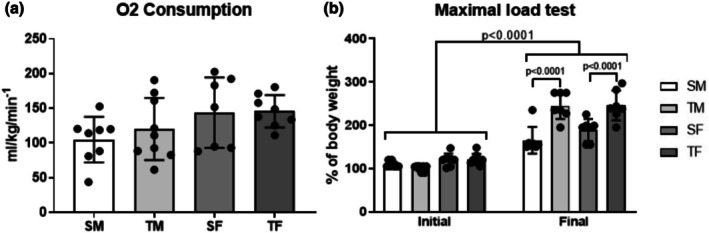
(a) Maximum oxygen consumption (VO_2_ max.) after 8 weeks of dynamic resistance training (*p* = 0.1412) and (b) Maximal load at the beginning (Initial) and after 8 weeks (Final) of the dynamic resistance training. SF, sedentary female; SM, sedentary male; TM, trained male; TF, trained female.

There were no significant differences in MBP between the groups (Table 1). There was no difference in VO_2_ max between the groups (Figure 2a, *p* = 0.1412). All groups showed an increase in maximal load at the end of the MLT protocol (*p* < 0.0001). However, the trained groups showed an even greater increase in maximal load than the sedentary groups (*p* < 0.0001) (Figure 2b).

**FIGURE 3:**
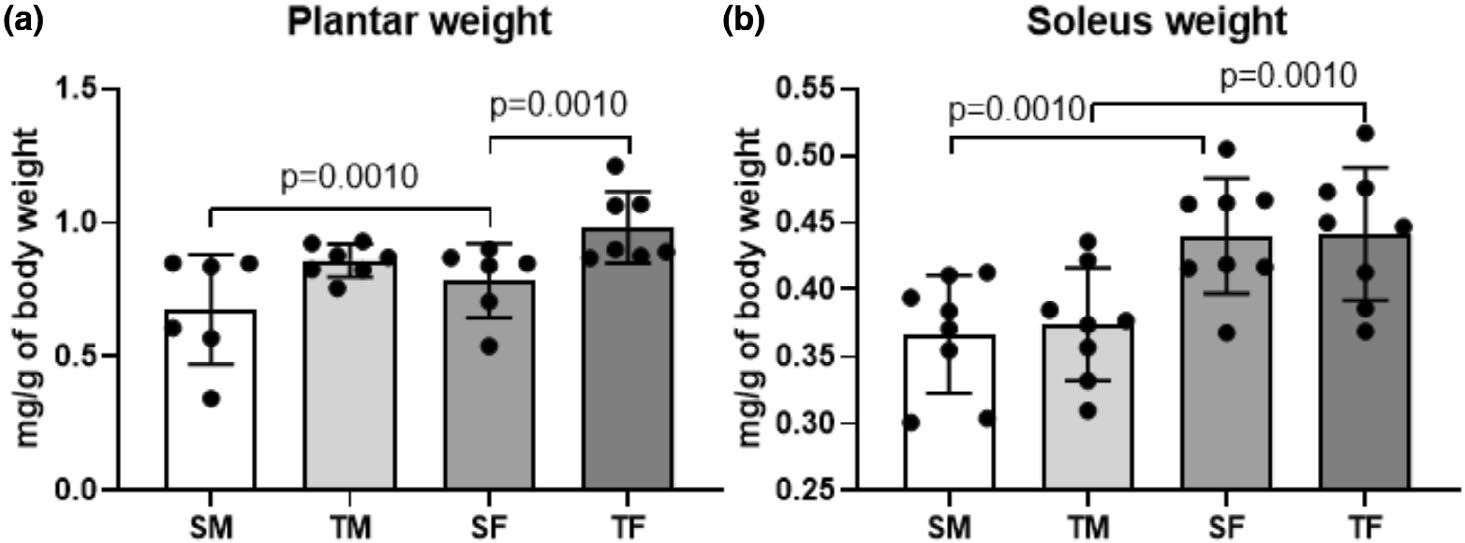
(a) Plantaris muscle weight and (b) soleus muscle weight after 8 weeks of dynamic the resistance training. SF, sedentary female; SM, sedentary male; TF, trained female; TM, trained male.

Both sexes in the trained groups showed increased plantaris muscle weight [(muscle weight/body weight)^100^] compared to their respective sedentary groups (Figure 3a, *p* = 0.0010). However, normalized soleus muscle weights were lower in males than in females (Figure 3b, *p* = 0.0010).

**FIGURE 4:**
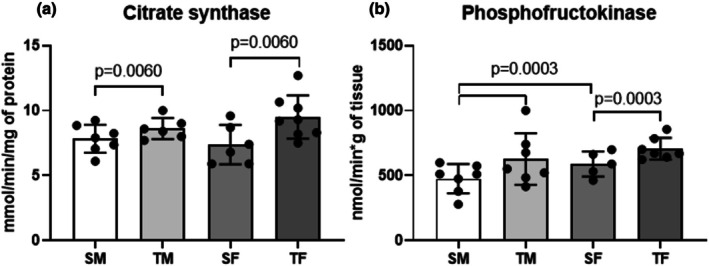
Citrate synthase activity in soleus muscle (*p* = 0.0060) and (b) Phosphofructokinase activity in plantaris muscle (*p* = 0.0003) after 8 weeks of the dynamic resistance training. SF, sedentary female; SM, sedentary male; TF, trained female; TM, trained male.

The trained groups of both sexes showed higher citrate synthase (CS) and phosphofructokinase (PFK) activities in the soleus and plantaris muscles, respectively, than the sedentary groups. Moreover, PFK activity in the plantaris muscle was higher in SF than in SM (Figure 4a,b).

**FIGURE 5:**
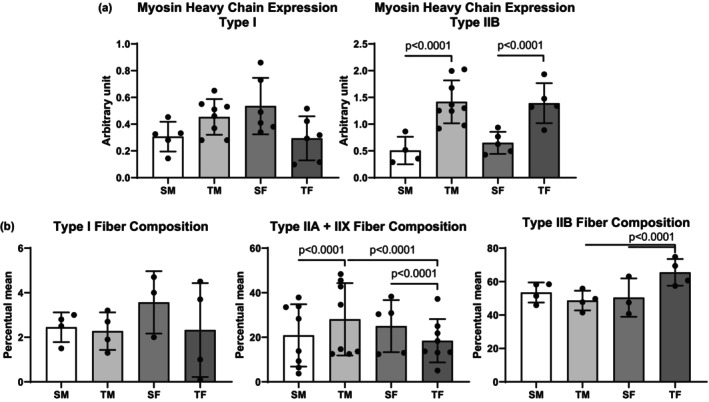
(a) Myosin heavy chain (MHC) expression in plantaris muscle and (b) plantaris muscle fiber composition by histochemical technique after 8 weeks of the dynamic resistance training. MHC I, myosin heavy chain type I; MHC IIB, myosin heavy chain type IIB; SF, sedentary female; SM, sedentary male; TF, trained female; TM, trained male.

No difference in MHC type I fibers was observed in the plantaris muscle between the studied groups. The trained groups of both sexes had more MHC IIB fibers than the sedentary groups (Figure 5a). Moreover, histological analysis showed that TF had fewer intermediate fibers (type IIA and IIX) than the SF group. However, TF had a higher percentage of type IIB fibers than SF and TM. Additionally, despite no change in type IIB fibers, TM had more intermediate fibers (types IIA and IIX) than SM and TF (Figure 5b).

**FIGURE 6:**
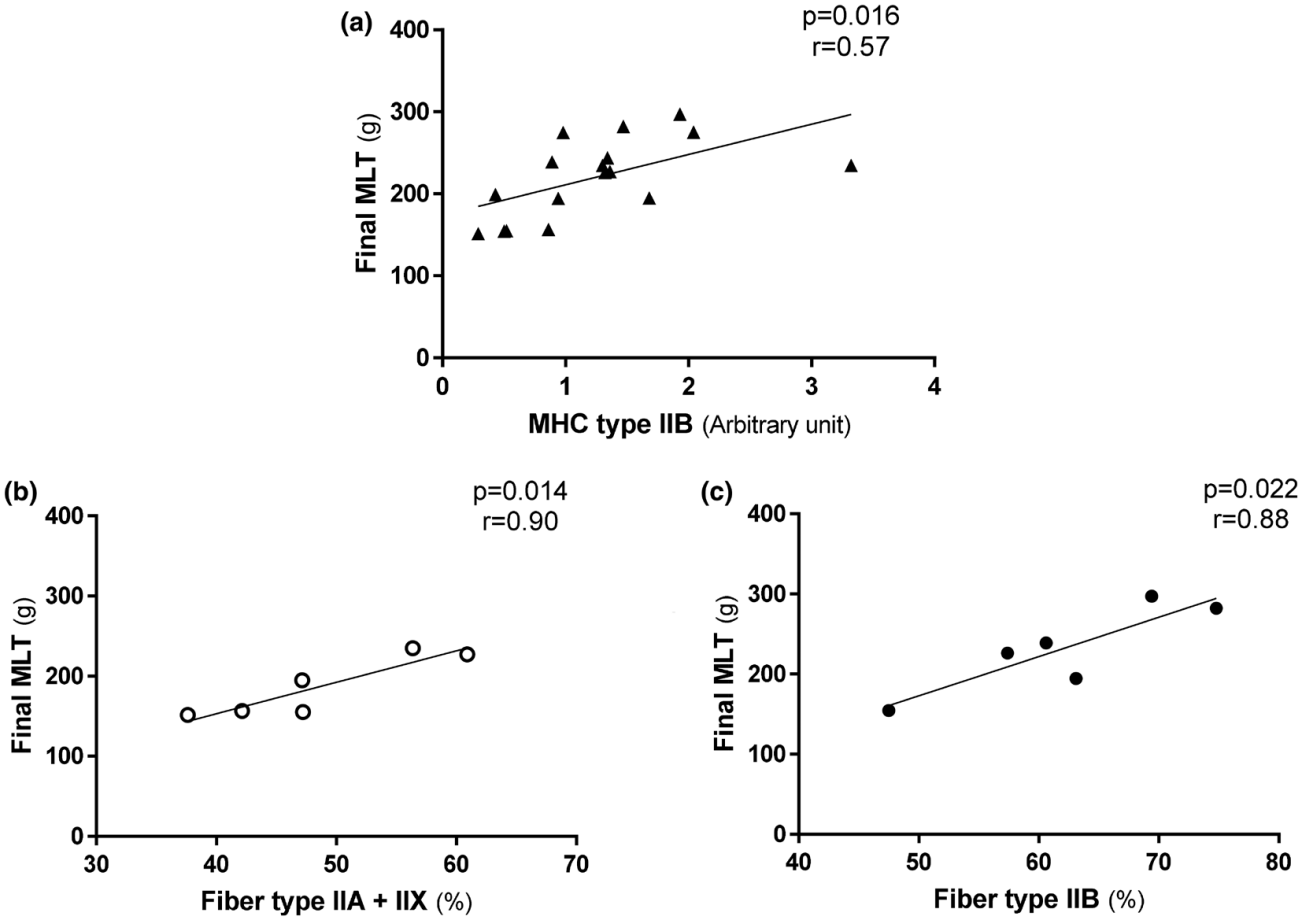
Correlation between: (a) Final maximal load test (MLT) and plantaris myosin heavy chain (MHC) type IIB expression including all groups (*r* = 0.71; *p* = 0.0003); (b) Final MLT and plantaris intermediate (IIA and IIX) fibers types for male groups (*r* = 0.83; *p* = 0.012) and (c) Final MLT and plantaris IIB fiber type for females groups (*r* = 0.74; *p* = 0.015).

We found a positive correlation between maximal load in the final test and MHC IIB expression in the plantaris muscle across all groups (Figure 6a, *r* = 0.57, *p* = 0.016). We also observed positive correlations between the final MLT and intermediate (IIA and IIX) fiber types in the plantaris muscle for the male groups (Figure 6b, *r* = 0.90; *p* = 0.014), and between the final MLT and IIB fiber type in the plantaris muscle for the female groups (Figure 6c, *r* = 0.88; *p* = 0.022).

## 4 DISCUSSION

Our main findings demonstrate that the applied training protocol elicited significant RT adaptations, reflected by improved MLT performance, increased PFK activity, and elevated MHC IIB expression in the plantaris muscle. Consistent with previous studies, our results also indicate that RT does not significantly affect maximal VO_2_ max or BP.

Despite considerable evidence suggesting that RT does not alter VO_2_ max, we showed that RT increased the enzymatic activity of citrate synthase, an important marker of oxidative metabolism, in the soleus muscle of trained groups regardless of sex. These results suggest that our RT protocol has a dynamic/aerobic component, which may contribute to the management of hypertension. Moreover, regarding sex differences, we demonstrated that female SHR, whether sedentary or trained, had greater normalized soleus muscle weight than did male SHR. In this sense, it is well known that women exhibit greater fatigue resistance in response to various fatigue protocols (Hicks et al., 2001), which could help explain this result given that the soleus muscle comprises mainly slow‐twitch or oxidative fibers.

However, it is important to note that our primary goal was to evaluate the anaerobic effects of the RT protocol in SHR. Accordingly, the trained groups of both sexes showed an increase in MLT performance at the end of the protocol, which indicates the effectiveness of this test and the RT performed in this study. Moreover, we observed differences in plantaris muscle weight between the sedentary and trained groups. It is worth noting that this muscle is mainly comprised of fast‐twitch fibers, and RT tends to activate this type of muscle fiber more effectively. These results are in accordance with previous data from our group showing an increase in MLT performance and plantaris weight gain with the same RT protocol (Sanches et al., 2014). Furthermore, we found a positive correlation between final MLT and plantaris MHC type IIB for all groups, which is another indicator of RT efficiency. Further evaluations of the metabolism of the plantaris muscle revealed increased PFK activity in the trained groups of both sexes. PFK is an important enzyme in glycolytic metabolism that reinforces the resistance/anaerobic characteristics of the training protocol performed in the current study. Thus, the moderate‐intensity dynamic RT protocol used in the present study contains both aerobic and anaerobic components, as suggested by Krüger et al. (2013), promoting beneficial adaptations in soleus and plantaris muscles in both sexes. However, the VO_2_ max remained unchanged; the final MLT increased significantly in the trained groups, indicating the dominant role of the anaerobic component of the RT protocol.

The present study also investigated the muscle fiber phenotype of the plantaris muscle to understand the effects of the RT in both male and female SHRs. Gene expression of MHC type IIB fiber was increased in the trained groups regardless of sex, reinforcing the characteristics of RT. However, analysis of fiber composition via histochemistry indicated that female rats adapted by increasing type IIB fibers, while male rats adapted by increasing intermediate fibers. It is important to note that type IIB and IIA fibers predominantly exhibit rapid contraction and glycolytic metabolism, which indicates the prevalence of the anaerobic RT component (Haizlip et al., 2015). However, type IIA and IIX fibers exhibit intermediate characteristics and have the potential to differentiate between anaerobic and aerobic fibers. This may represent sexual dimorphism in skeletal muscle, given that both sexes underwent the same training protocol in the current study and responded differently in terms of muscle histological composition. This finding provides a basis for future research to further explore this topic.

Considering the characteristics of each fiber type, it is reasonable to surmise that the same training intensity prescription does not produce the same physiological effects in both sexes. It is important to emphasize that evidence suggests men have more type IIB fibers than women, whether they are trained or sedentary. In contrast, women have a higher percentage of oxidative profile fibers (Horwath et al., 2021; Staron et al., 2000). However, there is no difference in the characteristics of motor units between the sexes (Miller et al., 1993). This suggests that the metabolic demand of this type of training may be greater for females than for males. Additionally, we note that numerous genes are regulated differently in the skeletal muscles of men and women, which can affect musculoskeletal structure (Welle et al., 2008). Therefore, an increase in intermediate fibers in males may indicate training with a greater dynamic component, as such fibers have greater fatigue tolerance despite being fast‐twitch and glycolytic. For females, who have a higher percentage of type IIB fibers in response to the same training, the exercise stimulus is greater for muscle power and strength, as these fibers have lower fatigue tolerance and contract faster than other fibers. This is related to power/explosive physical activities (Andersen & Aagaard, 2010). To support this idea, we found a positive correlation between final MLT and intermediate plantaris fibers in males, as well as between MLT and type IIB plantaris fibers in females. These results indicate that RT with the same intensity prescription (40%–60% of maximal load) promotes different histological adaptations according to sex.

Recent meta‐analyses have shown that, while men typically experience greater absolute increases in muscle strength and mass after RT, women often demonstrate equivalent or even greater relative improvements, especially in the upper limbs (Roberts et al., 2020). These findings suggest that the underlying mechanisms of adaptation, such as motor unit recruitment, hypertrophy, and metabolism, may be equivalent between sexes despite differences in magnitude. These observations echo our findings in SHRs, which showed qualitatively similar muscle adaptations, albeit with distinct histological patterns.

In conclusion, the present study characterized the skeletal muscle adaptations induced by dynamic RT in SHRs, taking into account sex‐related differences. Both male and female trained animals exhibited similar increases in enzymatic activity and muscle weight, indicating that RT promotes beneficial muscular adaptations regardless of sex. However, differences in muscle fiber‐type composition between sexes suggest the presence of sexual dimorphism in response to training, which should be considered in exercise prescription for hypertensive populations. Importantly, dynamic RT neither reduced nor exacerbated BP levels, suggesting it is a safe intervention for hypertensive individuals. The muscle‐specific responses observed in this study reinforce the potential of RT to enhance functional capacity, even in the absence of changes in BP. Further studies exploring the molecular pathways underlying muscle fiber remodeling in response to RT—with sex as a key biological variable—are warranted to better understand these adaptations and optimize nonpharmacological strategies for hypertension management.

## FUNDING INFORMATION

This study was supported by the São Paulo Research Foundation (FAPESP, grant no. 2015/10329‐5), the Coordination for the Improvement of Higher Education Personnel (CAPES, grant no. 88881.062178/2014‐01), and the National Council for Scientific and Technological Development (CNPq, grant nos. 407398/2021‐0 and 406792/2022‐4). The funding agencies had no role in study design, data collection and analysis, decision to publish, or preparation of the manuscript.

## CONFLICT OF INTEREST STATEMENT

None of the authors has any conflict of interest to disclose.

## ETHICS STATEMENT

We confirm that we have read the Journal's position on issues involved in ethical publication and affirm that this report is consistent with those guidelines.
